# Cytotoxicity and Apoptosis Induction of 6,7-Dehydroroyleanone from *Taiwania cryptomerioides* Bark Essential Oil in Hepatocellular Carcinoma Cells

**DOI:** 10.3390/pharmaceutics14020351

**Published:** 2022-02-02

**Authors:** Guan-Rong Chen, Mei-Ling Chang, Shang-Tzen Chang, Yu-Tung Ho, Hui-Ting Chang

**Affiliations:** 1 School of Forestry and Resource Conservation, National Taiwan University, Taipei 106, Taiwan; r01625035@ntu.edu.tw (G.-R.C.); peter@ntu.edu.tw (S.-T.C.); r10625032@ntu.edu.tw (Y.-T.H.); 2 Department of Food Science, Nutrition, and Nutraceutical Biotechnology, Shih Chien University, Taipei 104, Taiwan; mlchang@g2.usc.edu.tw

**Keywords:** apoptosis, cytotoxicity, hep g2 cell line, human hepatocellular carcinoma, *Taiwania cryptomerioides*

## Abstract

The objective of the present study is to evaluate the cytotoxicity of *Taiwania cryptomerioides* essential oil and its phytochemical on the Hep G2 cell line (human hepatocellular carcinoma). Bark essential oil has significant cytotoxicity to Hep G2 cells, and S3 fraction is the most active fraction in cytotoxicity to Hep G2 cells among the six fractions. The diterpenoid quinone, 6,7-dehydroroyleanone, was isolated from the active S3 fraction by bioassay-guided isolation. 6,7-Dehydroroyleanone exhibited significant cytotoxicity in Hep G2 cells, and the efficacy of 6,7-dehydroroyleanone was better than the positive control, etoposide. Apoptosis analysis of Hep G2 cells with different treatments was characterized via flow cytometry to confirm the cell death situation. Etoposide and 6,7-dehydroroyleanone could induce the apoptosis in Hep G2 cells using flow cytometric assay. Results revealed 6,7-dehydroroyleanone from *T. cryptomerioides* bark essential oil can be a potential phytochemical to develop the anticancer chemotherapeutic agent for the treatment of the human hepatocellular carcinoma.

## 1. Introduction

*Taiwania cryptomerioides* Hayata (Taxodaiceae) is one of the essential woody plants and distributed around Southeast Asia. *T. cryptomerioides*, *Ginkgo biloba*, *Sequoiadendron giganteum*, *Metasequoia glyptostroboides*, etc., are glacial relict plants and recognized as living fossil plants. Researchers have investigated the phytochemical and bioactivities of extracts from *T. cryptomerioides* heartwood and bark, which are rich in terpenoids and lignans. Previous studies have shown that natural products from *T. cryptomerioides* possess antibacterial, antifungal, anti-mite, mosquito larvicidal, anti-termite, antioxidant, anti-inflammatory, and antitumoral activities [1,2,3,4,5,6,7,8]. α-Cadinol, sesquiterpenoid separated from *T. cryptomerioides* heartwood, exhibited antiproliferative activity on Human colon adenocarcinoma cells [3]. Taiwanin A, the dibenzyl-γ-butyrolactone-type lignan, displayed superior antitumor activity against A-549 lung carcinoma and MCF-7 breast adenocarcinoma and colon adenocarcinoma cell line among the isolated lignans and sesquiterpenoids from *T. cryptomerioides* heartwood extract [1,9].

Hepatocellular carcinoma, primary liver cancer, is one of the main leading causes of cancer-related death in the world; major therapies are surgical resection, liver transplant, radio frequency ablation, chemotherapy, immunotherapy, etc. Medicinal drugs include sorafenib, lenvatinib, regorafenib, cabozantinib, and ramucirumab; hepatocellular carcinoma still lacks more effectual pharmacotherapies [10,11]. Many researchers are devoted to finding the promising chemicals from plant natural products for hepatocellular carcinoma treatment. *Camellia ptilophylla* (cocoa tea, naturally non-caffeinated tea) was found to possess anticancer and anti-obesity activities; green cocoa tea infusion could induce the apoptosis of Hep G2 cells [12]. Isoobtusilactone A, the phytochemical isolated from *Cinnamomum kotoense*, also presents a cytotoxic effect against Hep G2 cells [13]. Taxol (paclitaxel, a well-known chemotherapy drug derived from *Taxus brevifolia*) also inhibited the cytotoxicity in the HuH-7 cell line [14]. The growth of Hep G2 cells declined after being treated with β-thujaplicin in the xenograft model, and β-thujaplicin could induce both early- and late-stage apoptosis in Hep G2 cells [15]. The proliferation of Hep G2 cell line was inhibited by hinokiflavone; cell shrinkage, chromatin condensation, cell fragmentation, and apoptosis were observed in treated cell line [16].

The objectives of the present research are to evaluate the cytotoxicity and apoptosis induction of *T. cryptomerioides* bark essential oil in hepatocellular carcinoma cells and to find out its active phytochemical.

## 2. Materials and Methods

### 2.1. Hydrodistillation of Bark Essential Oil

Bark of *T. cryptomerioides*, around 40 years old, was collected from the Experimental Forest of National Taiwan University in Nantou County, Taiwan. The voucher specimen was kept in the laboratory of Chemical Utilization of Biomaterials, School of Forestry and Resource Conservation, National Taiwan University. Essential oil was extracted by hydrodistillation using the Clevenger apparatus for 8 h, the yield of bark essential oil was 0.32 mL/kg. Essential oil was stored in dark glass bottles at 4 °C [17].

### 2.2. Column Chromatography, Thin Layer Chromatography, and High-Performance Liquid Chromatography

Bark essential oil (8.10 g) was separated by silica gel column chromatography (CC) with the gradient elution of n-hexane and ethyl acetate of increasing polarity. Six fractions (S1–S6) were obtained by the analysis of thin layer chromatography (TLC) [18]. Yields of six fractions were S1 (4.18%), S2 (3.35%), S3 (57.48%), S4 (17.95%), S5 (13.52%), and S6 (3.52%). Active fraction, S3, were analyzed and separated by high-performance liquid chromatography (HPLC, L-2130, Hitachi, Tokyo, Japan) with a preparative Zorbax Sil column (250 mm × 9.4 mm, 5 μm). The gradient mobile phase consisted of n-hexane (A) and ethyl acetate (B). The flow rate was 2 mL/min. The elution program involved a linear gradient from 100% A for 0–3 min, 0 to 10% B in A by 3–25 min, 10 to 50% B in A by 25–35 min, and followed by 50% B in A for 10 min; the UV detector was operated at 254 nm [19,20].

### 2.3. Identification and Quantification of Isolated Compound

The chemical structure of isolated phytochemical was identified and characterized by spectral analyses, including UV/VIS (Ultraviolet-visible spectroscopy, V-550, Jasco, Tokyo, Japan), FTIR (Fourier transform infrared spectroscopy, FTS-40, Bio-rad, Hercules, CA, USA), and MS (mass spectroscopy, MAT-958, Finnigan, MA, USA). Additionally, 1D NMR (Nuclear magnetic resonance spectroscopy) (1H-NMR, 500 MHz; 13C-NMR, 125 MHz), and 2D NMR (HSQC, HMBC, COSY, and NOESY) were measured by the Bruker AVIII NMR spectrometer (Bruker Avance, Rheinstetten, Germany). The Oak Ridge Thermal Ellipsoid Plot (ORTEP) diagram was recorded with the X-ray single crystal diffractometer (XRD, SMART Apex CCD, Bruker, Karlsruhe, Germany) [21,22,23].

The quantification of the active compound was analyzed by a Thermo Trace GC Ultra gas chromatograph equipped with a Polaris Q MSD mass spectrometer (Thermo Fisher Scientific, Austin, TX, USA). Next, 1 μL analyte was injected into the DB-5MS capillary column (Crossbond 5% phenyl methyl polysiloxane, 30 m length × 0.25 mm i.d. × 0.25 µm film thickness). The temperature program was as follows: 60 °C initial temperature for 3 min; 3 °C/min up to 120 °C; 5 °C/min up to 240 °C and hold for 5 min. The flow rate of carrier gas, helium, was 1 mL/min, and the split ratio was 1:10. Compound was characterized by comparing the mass spectra (m/z 50–650 amu); quantification was analyzed by integrating the peak area of the chromatogram using the flame ionization detector (FID) [24,25].

### 2.4. Cell Culture and Cell Cytotoxicity Assay

Hep G2 cell, human hepatocellular carcinoma cell line, was provided by the Hepatitis Research Center (HRC) of the National Taiwan University Hospital (NTUH). Cell line was cultured in high-glucose DMEM (Dulbecco’s modified Eagle’s medium) supplemented with 10% FBS, 1% penicillin-streptomycin, and 1% NEAA (non-essential amino acid) at 37 °C in a humidified atmosphere with 5% CO_2_. For cell cytotoxicity assay, Hep G2 cells were seeded in a 96 well plate (2 × 104 cell/well) and incubated for 24 h, then the medium was removed and treated with various concentrations of specimens in 0.2% of DMSO (dimethyl sulfoxide) for 24 h. After the medium was removed, MTT (3-(4,5-dimethylthiazol-2-yl)-2,5-diphenyl tetrazolium bromide), 1 mg/mL) reagent was added in each well for 1 h, then the medium containing MTT was removed, and 100 µL of DMSO was added to the well to solubilize the formazan crystals. Absorbance was measured at 570 nm using an ELISA (enzyme-linked immunosorbent assay) reader (SPECTROstar Nano, BMG LABTECH, Offenburg, Germany). The percentage of cytotoxicity was calculated by the following formula: Cytotoxicity (%) = (OD_control_ − OD_sample_)/OD_control_ × 100. The experiments were performed in triplicate [26,27,28].

### 2.5. Apoptosis Analysis Using Flow Cytometric Assay

**Figure 1:**
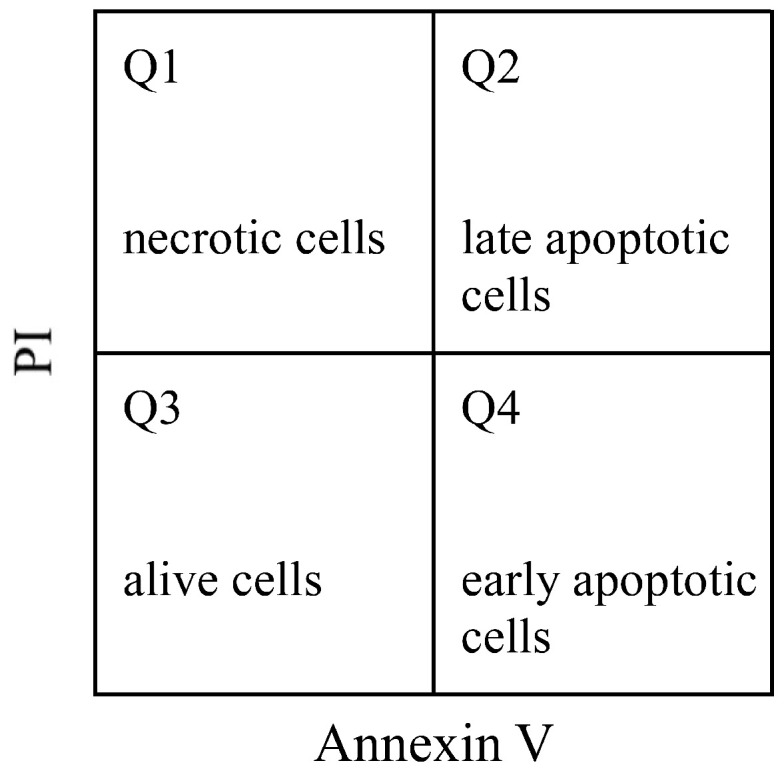
Differentiation of four cell populations by double immunocytochemistry labeling.

Hep G2 cells were seeded in a 6-well plate (2 × 105 cell/well) and incubated for 24 h, then the medium was removed and replaced with the medium containing various concentrations of specimens for 4, 24, and 48 h. Cells were collected and washed twice with PBS, then suspended in 500 μL of Annexin binding buffer containing 5 µL of Alexa fluor^®^ 488 Annexin V and 5 µL of PI (propidium iodide) for 15 min in the dark. The apoptotic effect of Hep G2 cells treated with different specimens was analyzed using Cytomics FC500 flow cytometry (Beckman Coulter, Miami, FL, USA). Double immunocytochemistry labeling with FL1 (Annexin V; green fluorescence) and FL3 (propidium iodide; red fluorescence) was used to differentiate four populations (four quadrants in Figure 1), including necrotic cells (Q1, red fluorescent), late apoptotic cells (Q2, both green/red fluorescent), alive/healthy cells (Q3, no fluorescent), and early apoptotic cells (Q4, green fluorescent) [29,30].

### 2.6. Statistical Analysis

The statistical analysis was performed by using SPSS (Statistical Product and Service Solutions) (Chicago, IL, USA) version 16 with Scheffe’s test, a post hoc multiple comparison method. The confidence interval was set at the 95% confidence level.

## 3. Results and Discussion

### 3.1. Cytotoxic Effect of T. cryptomerioides Bark Essential Oil and Its Fractions on Hep G2 Cells

The cytotoxicity and IC_50_ value of *T. cryptomerioides* bark essential oil and its fractions against Hep G2 cells were shown in Table 1. The antiproliferation of bark essential oil was increased with the concentration. The cytotoxicity of bark essential oil was 77.80% at a concentration of 100 µg/mL against Hep G2 cells; the IC_50_ value was 54.31 μg/mL for 24 h. Myint et al. investigated the cytotoxic effect of *Smallanthus sonchifolius* leaf extract on Hep G2 cells. The IC_50_ value of leaf extract was 58.2 µg/mL after 24 h treatment [31]. The green cocoa tea (*Camellia ptilophylla*) infusion possessed the antiproliferation of Hep G2 cells in a dose-dependent effect with an IC_50_ value of 292 μg/mL for 72 h [12]. Results from the MTT assay revealed that *T. cryptomerioides* bark essential oil had a potent cytotoxic effect on Hep G2 cells.

Among the six fractions obtained from open column chromatography of bark essential oil, S2 fraction and S3 fraction possessed a better cytotoxic effect on Hep G2 cells. The IC_50_ values of the other fractions were greater than 50 μg/mL. The IC_50_ values of S2 fraction and S3 fraction were 32.22 and 21.17 μg/mL for 24 h, respectively (Table 1). S3 fraction showed the best cytotoxicity in Hep G2 cells with a statistical significance of *p* < 0.05.

### 3.2. Identification of Chemical and Molecular Structures of 6,7-Dehydroroyleanone

**Figure 2:**
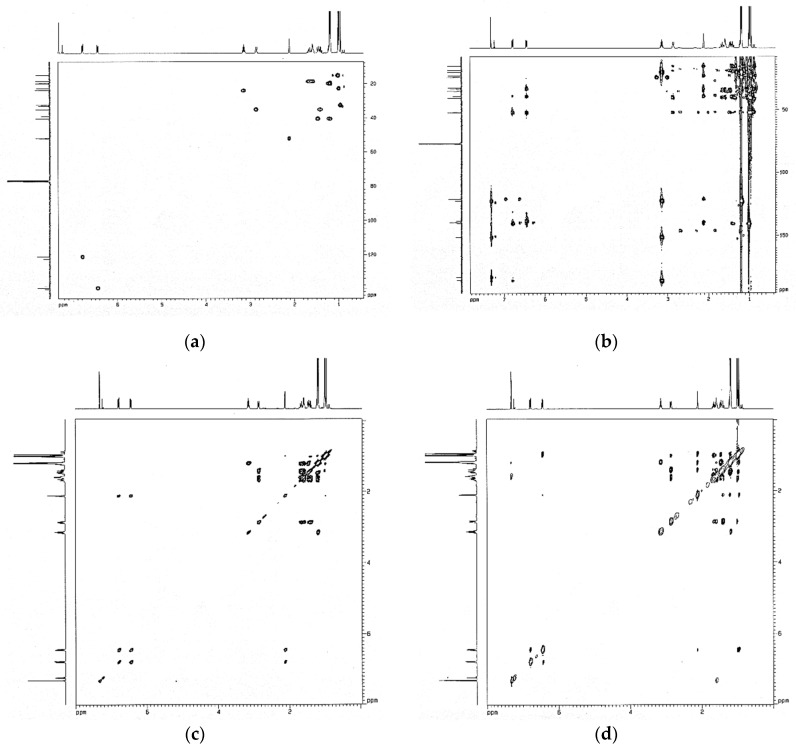
2D NMR spectra of 6,7-dehydroroyleanone. (**a**) HMQC; (**b**) HMBC; (**c**) COSY; (**d**) NOESY.

**Figure 3:**
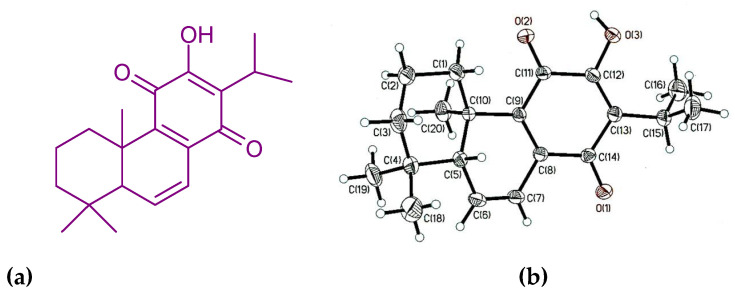
Chemical and molecular structures of 6,7-dehydroroyleanone. (**a**) Chemical structure; (**b**) ORTEP diagram of the molecular structure.

The structure of the compound from active fraction S3 is elucidated based on the 1D and 2D NMR (Figure 2), single-crystal XRD, FTIR, UV, and MS spectroscopic analyses. 6,7-Dehydroroyleanone 232 mg. Orangish red needle; mp: 168–169 °C; UV (MeOH) *λ*_max_ (log ε): 221.0 (3.72), 251.0 (3.51), 333.5 (3.43), and 461.5 (2.43) nm; [α]_D_^21.8^ = −248.0° (CHCl_3_; c = 0.13); IR (KBr) *ν*_max_ 3364, 3089, 2961, 2927, 2870, 1626, 1551, 1460, 1387, 1376, 1329, 1254, and 1165 cm^−1^; 1H NMR (CDCl_3_, 500 MHz) *δ* 7.32 (1H, *s*, OH-12), 6.78 (1H, *dd*, *J* = 9.8, 3.0 Hz, H-7), 6.44 (1H, *dd*, *J* = 9.8, 3.0 Hz, H-6), 3.14 (1H, *hept*, *J* = 7.0 Hz, H-15), 2.86 (1H, *d*, *J* = 13.4 Hz, H-3a), 2.12 (1H, *t*, *J* = 3.0 Hz, H-5), 1.67 (1H, *m*, H-2a), 1.59 (1H, *m*, H-2b), 1.47 (1H, *d*, *J* = 13.4 Hz, H-1a), 1.41 (1H, *td*, *J* = 13.4, 3.8 Hz, H-3b), 1.23 (1H, *m*, H-1b), 1.20 (3H, *d*, *J* = 7.0 Hz, H-17), 1.19 (3H, *d*, *J* = 7.0 Hz, H-16), 1.01 (3H, *s*, H-19), 0.99 (3H, *s*, H-18), 0.96 (3H, *s*, H-20). 13C NMR (CDCl_3_, 125 MHz) *δ* 186.06 (C-14), 183.44 (C-11), 151.19 (C-12), 140.51 (C-8), 139.6 (C-6), 138.5 (C-9), 122.59 (C-13), 121.10 (C-7), 52.10 (C-5), 40.52 (C-1), 39.25 (C-4), 35.15 (C-3), 33.26 (C-10), 32.60 (C-20), 24.08 (C-15), 22.80 (C-18), 20.00 (C-16), 19.80 (C-17), 18.67 (C-2), 15.17 (C-19). EI-MS m/z 314 [M]^+^, 299, 271, 258, 245, 232, 213, 187, 159, 141, 128, 115, 83, molecular formula C_20_H_26_O_3_; single-crystal XRD analysis: monoclinic crystal system; P2_1_ space group. The chemical structure and ORTEP diagram of the identified compound are shown in Figure 3; 6,7-dehydroroyleanone is a diterpenoid quinone based on an abietane skeleton. Quantification of compound 6,7-dehydroroyleanone was 8.81 ± 0.21% using the analysis of GC.

6,7-Dehydroroyleanone has been isolated from *Inula royleana* root, *Plectranthus grandidentatus*, *P. madagascariensis*; *Salvia lavandulaefolia* root, *Salvia jaminiana* root, *Taxodium distichum* cone and seed, *Tetradenia riparia* leaf [32,33,34,35,36,37,38,39,40]. The bioactivities of 6,7-dehydroroyleanone have been reported, including anti-termite, antioxidant, antimicrobial, antileishmanial, and anti-Mycobacterium tuberculosis activities [36,37,38,41,42,43]. 6,7-Dehydroroyleanone exhibits the cytotoxicity activity against breast cancer cell lines, including MCF-7 (hormone-positive breast cancer cells), SkBr3 Her-positive, SUM159 triple-negative, and SUM159 spheres [44], and primary H7PX glioma cell lines [45].

### 3.3. Cytotoxic Effect of 6,7-Dehydroroyleanone on Hep G2 Cells

Etoposide, a podophyllotoxin derivative, exhibits topoisomerase II inhibition activity, thereby leading to the cytotoxicity of tumor cells [46]. Etoposide was selected as the cytotoxicity positive control in the current study. The cytotoxic activities of etoposide, bark essential oil, and 6,7-dehydroroyleanone against Hep G2 cells were presented in Table 2. The IC_50_ values of etoposide were 76.89 μg/mL (130.64 μM) and 29.68 μg/mL (51.84 μM) for 24 and 48 h. Bakherad et al. reported that etoposide exhibited cytotoxicity against Hep G2 cells with an IC_50_ value of 31.38 μM after 48 h incubation [47]; the activity of etoposide was similar to that of the present study.

Bark essential oil exhibited cytotoxicity against Hep G2 as potent as etoposide; the IC_50_ values were 30.27 and 29.68 µg/mL for 48 h treatment, respectively; there were no statistically significant differences (*p* < 0.05) in the MTT assay. The cytotoxicity of 6,7-dehydroroyleanone was more effective than those of bark essential oil and etoposide. The IC_50_ values of 6,7-dehydroroyleanone were 10.28 μg/mL (32.74 μM) and 5.22 μg/mL (16.62 μM) for 24 and 48 h. Results revealed that 6,7-dehydroroyleanone is a promising phytochemical which possesses a significant superior cytotoxic effect on Hep G2 cells, of which the efficacy was superior to the positive control, etoposide.

### 3.4. Apoptosis Effect of 6,7-Dehydroroyleanone on Hep G2 cells

**Figure 4:**
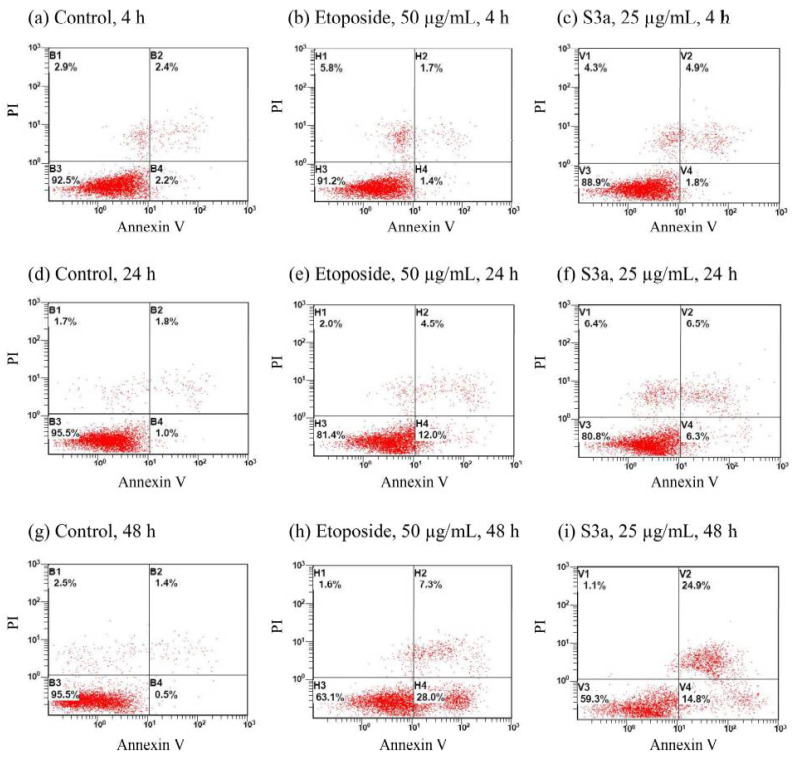
Apoptosis analysis by Annexin V/PI flow cytometry of treated Hep G2 cells. (**a**) Control, 4 h; (**b**) etoposide, 50 µg/mL, 4 h; (**c**) S3a, 25 µg/mL, 4 h; (**d**) control, 24 h; (**e**) etoposide, 50 µg/mL, 24 h; (**f**) S3a, 25 µg/mL, 24 h; (**g**) control, 48 h; (**h**) etoposide, 50 µg/mL, 48 h; (**i**) S3a, 25 µg/mL, 48 h. S3a: 6,7-dehydroroyleanone.

**Figure 5:**
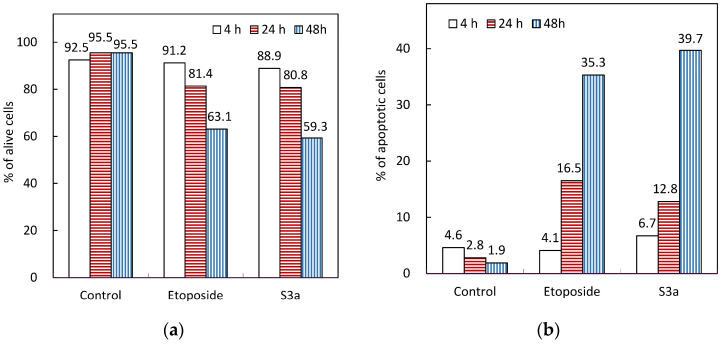
Etoposide and 6,7-dehydroroyleanone induced apoptosis in Hep G2 cells. (**a**) alive cells; (**b**) apoptotic cells. Etoposide, 50 µg/mL; S3a: 6,7-dehydroroyleanone, 25 µg/mL.

The apoptosis effects of etoposide and 6,7-dehydroroyleanone on Hep G2 cells were evaluated by using an Annexin V/propidium iodide staining method (Figure 4). The percentage of alive cells (Q2 area) of control were above 92% during the treatment period. Content of the apoptotic cells of control were kept below five percent during the 48 h of treatment. The necrotic cells (Q1 area) of the Hep G2 cell treated with etoposide was 5.8% for 4 h of treatment at a concentration of 50 μg/mL; a decrease in the necrotic process was observed after 24 and 48 h of treatment. Both Hep G2 cells exposed to etoposide and 6,7-dehydroroyleanone for 48 h had undergone the early and late apoptotic process (Q4 and Q2 areas), programmed cell death. The percentage of apoptotic cells of cells was 35.3 and 39.7% after 48 h of treatment with etoposide and 6,7-dehydroroyleanone, respectively (Figure 5). The apoptotic cells of all treated cells increased with the incubation time. Apoptosis effect of 6,7-dehydroroyleanone was confirmed through the Annexin V/PI flow cytometry assay. Sitarek et al. also reported that 6,7-dehydroroyleanone possessed the antiproliferative effect in H7PX glioma cell line by apoptosis; a similar anticancer effect was observed in this study [45]. Taiwanin A, a lignan isolated and identified from *T. cryptomerioides* heartwood extract, could also induce apoptosis in Hep G2 cells [6].

## 4. Conclusions

Using the colorimetric MTT assay, *T. cryptomerioides* bark essential oil and S3 fraction show the high cytotoxicity to Hep G2 cells. 6,7-Dehydroroyleanone was isolated from the fraction that possessed the best cytotoxicity through bioassay-guided fractionation; it is classified as a diterpenoid quinone determined by spectroscopic analyses. 6,7-Dehydroroyleanone exhibited better cytotoxicity in Hep G2 cells than etoposide, a clinical antitumor drug. The cytotoxicity of etoposide, bark essential oil, and 6,7-dehydroroyleanone was in a dose-dependent manner against the Hep G2 cells. Apoptosis analysis indicated that etoposide and 6,7-dehydroroyleanone induce apoptosis in Hep G2 cells, including early and late programmed cell death. These findings in the current study revealed 6,7-dehydroroyleanone from *T. cryptomerioides* bark essential oil merits further research and trials to develop as a chemotherapeutic phytochemical for human hepatocellular carcinoma.

## Figures and Tables

**Table 1:** Cytotoxicity of bark essential oil and active fractions against Hep G2 cells.

Specimen	Concentration (μg/mL)	Cytotoxicity * (%)	IC_50_ * (μg/mL)
Bark oil	12.5	10.23 ± 0.98	54.31 ± 0.60 ^a^
	25	22.20 ± 0.10	
	50	49.47 ± 0.17	
	100	77.80 ± 0.17	
S2 fraction	12.5	18.81 ± 0.90	32.22 ± 4.84 ^b^
	25	26.43 ± 9.70	
	50	84.44 ± 1.75	
	100	90.62 ± 0.33	
S3 fraction	12.5	44.37 ± 4.50	21.17 ± 1.09 ^c^
	25	55.98 ± 3.57	
	50	82.61 ± 0.70	
	100	93.38 ± 0.23	

Results are mean ± SD; *: 24 h of treatment; IC_50_ value: half maximal inhibitory concentration. Different letters (a–c) in the Table indicate significantly different at the level of *p* < 0.05 according to Scheffe’s test.

**Table 2:** Cytotoxicity of bark essential oil and 6,7-dehydroroyleanone against Hep G2 cells.

Time (h)	Specimen	IC_50_ (μg/mL)	IC_50_ (µM)
24	Bark essential oil	54.31 ± 0.60 ^b^	-
	6,7-Dehydroroyleanone	10.28 ± 0.18 ^d^	32.74 ± 0.57 ^C^
	Etoposide *	76.89 ± 0.34 ^a^	130.64 ± 0.58 ^A^
48	Bark essential oil	30.27 ± 1.11 ^c^	-
	6,7-Dehydroroyleanone	5.22 ± 0.09 ^e^	16.62 ± 0.29 ^D^
	Etoposide *	29.68 ± 1.18 ^c^	50.43 ± 2.01 ^B^

Results are mean ± SD. *: Positive control. IC_50_ value: half maximal inhibitory concentration. Different letters (a–e; A–D) in the Table indicate significant difference at the level of *p* < 0.05 according to Scheffe’s test.

## Data Availability

The data are available are available from the corresponding author on reasonable request.
